# THE IMPACT OF SPATIAL SCALE AND HABITAT CONFIGURATION ON PATTERNS OF TRAIT VARIATION AND LOCAL ADAPTATION IN A WILD PLANT PARASITE

**DOI:** 10.1111/evo.12239

**Published:** 2013-09-11

**Authors:** Ayco J M Tack, Felix Horns, Anna-Liisa Laine

**Affiliations:** 1 Metapopulation Research Group, Department of Biosciences, University of HelsinkiPO Box 65 (Viikinkaari 1), University of Helsinki, FI-00014, Finland

**Keywords:** Epidemiology, genotype-by-genotype interactions, habitat fragmentation, host–parasite interactions, metapopulation, spatial context

## Abstract

Theory indicates that spatial scale and habitat configuration are fundamental for coevolutionary dynamics and how diversity is maintained in host–pathogen interactions. Yet, we lack empirical data to translate the theory to natural host–parasite systems. In this study, we conduct a multiscale cross-inoculation study using the specialist wild plant pathogen *Podosphaera plantaginis* on its host plant *Plantago lanceolata*. We apply the same sampling scheme to a region with highly fragmented (Åland) and continuous (Saaremaa) host populations. Although theory predicts higher parasite virulence in continuous regions, we did not detect differences in traits conferring virulence among the regions. Patterns of adaptation were highly scale dependent. We detected parasite maladaptation among regions, and among populations separated by intermediate distances (6.0–40.0 km) within the fragmented region. In contrast, parasite performance did not vary significantly according to host origin in the continuous landscape. For both regions, differentiation among populations was much larger for genetic variation than for phenotypic variation, indicating balancing selection maintaining phenotypic variation within populations. Our findings illustrate the critical role of spatial scale and habitat configuration in driving host–parasite coevolution. The absence of more aggressive strains in the continuous landscape, in contrast to theoretical predictions, has major implications for long-term decision making in conservation, agriculture, and public health.

The strength and outcome of coevolutionary interactions is highly variable across space and time, ranging from hotspots with rapid reciprocal coevolution to coldspots where the two species do not coevolve (Laine 2009; Thompson 2013). Given such variable outcomes of coevolutionary interactions, the original question of whether natural selection plays a key role in host–parasite dynamics has recently shifted toward the question of when—and under what circumstances—we are most likely to witness evolutionary responses (Hereford 2009; Tack and Roslin 2010; Thompson 2013). Although the outcome of coevolution is generally expected to depend on the balance between selection, drift, and gene flow (Slatkin 1987; Lenormand 2002), few studies have explored how the relative strength of these factors—and hence the outcome of natural selection—depends on the spatial scale of the study and the configuration of the habitat. For example, although the meta-analytical approach has pinpointed several characteristics of the study system that may affect the strength of local adaptation (e.g., generalist vs. specialist or sessile vs. mobile parasites; Lajeunesse and Forbes 2002; Greischar and Koskella 2007; Hoeksema and Forde 2008), such an approach often ignores the fact that patterns of local adaptation may vary within a single species or community (Laine 2005; Tack and Roslin 2010). As a consequence, there is a clear need for evolutionary studies replicating experiments within a single pathosystem across spatial scales and across landscapes that differ in the configuration of the habitat.

Although few researchers have replicated local adaptation studies across multiple spatial scales within a single host–parasite system (Hanks and Denno 1994; Mopper et al. 1995; Thrall et al. 2002; Laine 2005), a few general patterns emerge from the studies to date. In a pioneering study, Mopper et al. (1995) demonstrated that local adaptation of a lepidopteran leafminer occurred at scales ranging from individual oak trees to oak populations separated by 65 km. In contrast, Laine (2005) demonstrated the presence of local adaptation of the powdery mildew *Podosphaera plantaginis* to its host plant *Plantago lanceolata* at the scale of tens of kilometers, whereas the pathogen showed no consistent pattern of adaptation at scales ranging from a few hundred meters to several kilometres. As the pathogen frequently dispersed up to a kilometer, the author suggested that local adaptation of the pathogen was swamped by gene flow at this small spatial scale. Corroborating this result, a cross-species comparison showed that plants are least resistant to local plant parasites and are most resistant to parasites collected several tens to hundreds kilometres away (Laine et al. 2011). However, the lack of gene flow among widely separated populations may also prevent adaptation at large spatial scales: whereas local hen flea populations were maladapted to local great tit populations as compared to nonlocal great tit populations on the same island (3.8–28.5 km between populations; Lemoine et al. 2012), another study did not find evidence for either local adaptation or maladaptation of flea populations separated by about 300 km (Dufva 1996). Overall, these studies may indicate that local adaptation is most likely to occur at “intermediate” spatial scales— where the definition of “intermediate” will depend on the balance between gene flow, relative dispersal ability of host and parasite, and the strength of natural selection (Gandon et al. 1996; Gandon 2002; Gandon and Michalakis 2002).

At any particular spatial scale, the evolutionary outcome of host–parasite interactions may strongly depend on habitat configuration (i.e., the spatial distribution of the habitat). Indeed, several theoretical studies have demonstrated the impact of habitat configuration on rapid and directional trait evolution (Rand et al. 1995; Boots and Sasaki 1999, 2000; Haraguchi and Sasaki 2000; Keeling 2000; O'Keefe and Antonovics 2002; van Baalen 2002; Boots et al. 2004; Kamo et al. 2007; Wild et al. 2009; Lion and Boots 2010; Best et al. 2011), trait diversity (Carlsson-Granér and Thrall 2002; Gandon and Michalakis 2002; Thrall and Burdon 2002; Kamo et al. 2007; Best et al. 2011), and local adaptation (Gandon et al. 1996; Gandon 2002; Gandon and Michalakis 2002) in host–parasite interactions. Although each of these models assesses trait evolution in a spatial perspective, the assumptions and ways of incorporating space vary widely (Lion and Boots 2010; Webb et al. 2013). For example, several theoretical studies investigate the impact of local and global dispersal or transmission on parasite evolution within a spatially substructured population, which generally leads to the prediction that virulence will decrease with more localized dispersal or transmission (Boots and Sasaki 1999; Haraguchi and Sasaki 2000; Best et al. 2011). However, as the majority of these studies do not consider host evolution (but see Best et al. 2011), they may not be suitable for deriving predictions when reciprocal evolution drives host–parasite dynamics. Coevolutionary models generally focus on the evolution of qualitative gene-for-gene interactions in a metapopulation characterized by infrequent dispersal among populations, and emphasize the general aspect that trait diversity can be maintained within metapopulations (Gandon et al. 1996; Nuismer et al. 2000; Thrall and Burdon 2002; Laine and Tellier 2008; Brown and Tellier 2011). Notably, specific outcomes may be affected by model assumptions including parasite life-history (e.g., O'Keefe and Antonovics 2002) and the postulation of trade-offs (and their shape) between parasite life-history traits (Anderson and May 1982; Kamo et al. 2007; Webb et al. 2013).

Although the theoretical prediction that parasite virulence, aggressiveness and diversity may evolve in response to changes in habitat configuration and increasing human movements are highly relevant for public health, agriculture, and conservation (Galvani 2003), theory has largely outpaced empirical studies in this field of research. A potential reason is the lack of any clear linkage between host–parasite systems as envisaged in silico and as observed in nature. In particular, the discrepancy between model assumptions and the complexity of real parasite life histories makes it challenging to summarize the diverse model outcomes and make a priori predictions for any specific natural host–parasite system. Nonetheless, two microevolutionary selection experiments have successfully validated model predictions on parasite trait evolution. Boots and Mealor (2007) showed that a high viscosity of the landscape (with resulting low movement rates and increased local interactions of the larvae of the moth *Plodia interpunctella*) selected for lower infectivity of a species-specific granulosis virus (PiGV). Kerr et al. (2006) found that localized dispersal in a phage-bacterial system increased dominance of competitively restrained “prudent” phage morphs, whereas “rapacious” phage evolved under unrestricted migration.

Even fewer studies have investigated the impact of the spatial configuration of the habitat on local adaptation. In one example, Tack and Roslin (2010) demonstrated that leaf miners and gallers were locally adapted to individual oak trees when immigration from neighboring trees was relatively low, whereas the insect community was nonadapted or maladapted when immigrants formed a large fraction of the local population. A bacteria-phage experiment further demonstrated that the shape of spatial dispersal networks may play a role in driving host–parasite coevolution and patterns of local adaptation (Vogwill et al. 2010). These studies then indicate that the configuration of the habitat, which provides the blueprint for gene flow across the landscape, may play a key role in host–parasite coevolution and local adaptation.

Finally, few studies have compared patterns of genetic and phenotypic differentiation among populations. In principle, such a comparison may reveal the spatial scale and type of natural selection (Merilä and Crnokrak 2001; Jorgensen et al. 2006; Tack et al. 2012). For example, if the main part of phenotypic diversity occurs within populations, whereas populations are genetically differentiated, this may indicate the maintenance of phenotypic trait variation by balancing selection within populations. In contrast, divergent selection would result in large phenotypic differentiation among populations as compared to genetic differentiation among populations.

In this article, we investigate the impact of both spatial scale and habitat configuration on parasite local adaptation of the powdery mildew *P. plantaginis* to its host plant *P. lanceolata*. Local adaptation, measured as higher parasite fitness on sympatric versus allopatric plants is taken as evidence for on-going coevolution (for other measurements of local adaptation, see Kawecki and Ebert 2004). Specifically, we investigate patterns of local adaptation and trait variation across three spatial scales: (i) among populations situated less than 1.6 km apart; (ii) among populations spaced 6–40 km apart; and (iii) among two regions (Åland and Saaremaa) set about 200 km apart and separated by a large body of water (Fig. 1). We employed an identical sampling scheme in both regions by collecting hosts and parasites at the same distances. As the regions differ in terms of the spatial configuration of the host populations (with Åland characterized by fragmented host populations, and Saaremaa by large continuous host populations), this design allows us to simultaneously test for impacts of habitat configuration on patterns of mean levels of phenotypic traits, trait diversity, and local adaptation.

**Figure 1:**
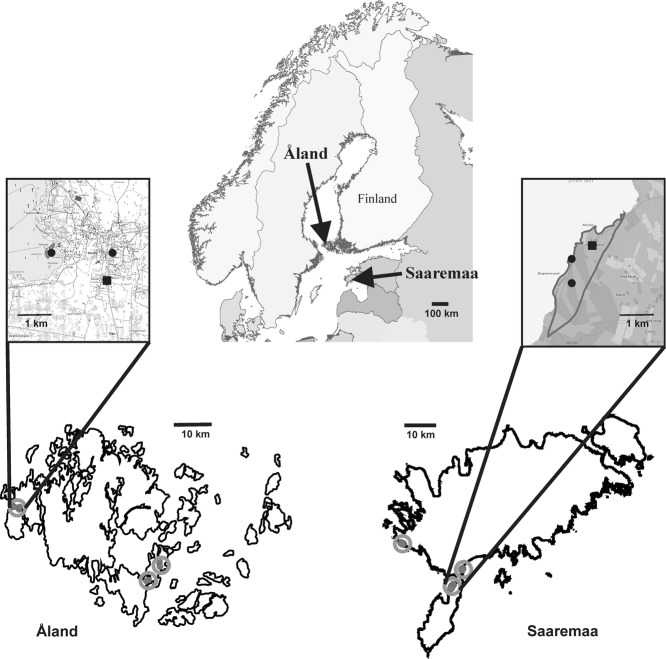
Map of sampling locations. The large upper panel shows a map of northern Europe with the location of the two island systems (Åland and Saaremaa) indicated by arrows. The lower panels reflect Åland (left) and Saaremaa (right), with the clusters shown by circles and populations by dots within these circles. For each region, a single cluster is shown in detail, with the distribution of the host indicated by a black outline and the sampling locations by filled black squares and circles (to indicate focal and nonfocal populations, respectively; note that the black squares and circles partly overlap with the host distribution outline in Åland).

In an attempt to bridge the gap between the theoretical literature and empirical studies, we put forward a selection of a priori hypotheses derived from modeling work but hardly tested in natural systems:

1. *The scale of local adaptation*. Local adaptation is expected to depend on the balance between selection and gene flow (Slatkin 1987; Lenormand 2002). Hence, we expect to find the strongest local adaptation at an intermediate spatial scale, where local adaptation is not swamped by gene flow, but interactions and movement are frequent enough for natural selection to play a role. Given the large dispersal range of aerially dispersed plant pathogens (Brown and Hovmøller 2002), we expect that “intermediate” distances may range from about 10 km to several hundreds of kilometres.
2. *Effects of habitat configuration on*:

- *Mean trait values*. Lower pathogen virulence will prevent overexploitation in small populations (“self-shading” or “kin shading”) without displacement by the more aggressive pathogen strain (Rand et al. 1995; Boots and Sasaki 1999, 2000; Haraguchi and Sasaki 2000; Keeling 2000; O'Keefe and Antonovics 2002; Kamo et al. 2007; Wild et al. 2009; Best et al. 2011). From the plant perspective, clustering of the resistant host and its offspring will increase the benefit of higher host resistance (Best et al. 2011). Basically, these arguments go back to Hamilton's (1964) classic conjecture that spatial structure is beneficial to cooperation, because cooperators can gain additional benefits from being clustered (see also Lion and van Baalen 2008). Hence, we expect that pathogen aggressiveness, which is commonly correlated with virulence, will be lower in the fragmented populations of Åland than in the continuous populations of Saaremaa.
- *Trait diversity*. Nonspatial host–parasite models predict that coevolutionary dynamics and cycles may result in the loss of phenotypic variation in a single mixed population (e.g., Leonard 1977). Subsequent models have shown that spatial structure may increase trait diversity for qualitative traits like gene-for-gene interactions during infection (Thrall and Burdon 2002; Laine and Tellier 2008; Brown and Tellier 2011). Hence, we expect higher trait diversity in the fragmented region than in the continuous region.
- *Local adaptation*. Previous studies detected parasite local adaptation in our study system (Laine 2005, 2008). We then expect that the high extinction rates of parasite populations and population bottlenecks in the fragmented region are likely to wipe out or weaken local parasite adaptation (Bergstrom et al. 1999; Mopper et al. 2000). Hence, we expect stronger local adaptation in the continuous than in the fragmented region.

(3) *Genetic versus phenotypic differentiation among populations*. Coevolutionary models and (more sparse) empirical data predict that negative frequency-dependent selection will maintain phenotypic diversity at a small spatial scale. This pattern is expected due to adaptation of the parasite to the most common local host genotypes and *vice versa* (Haldane 1949; Chaboudez and Burdon 1995; Dybdahl and Lively 1998; Lively and Dybdahl 2000; Brown and Tellier 2011). As neutral genetic variation is unaffected by such balancing selection, we may then expect more population differentiation among presumptively neutral genetic marker loci than among phenotypic traits.

## Materials and Methods

### STUDY SYSTEM

The powdery mildew *P. plantaginis* (Castagne; U. Braun and Takamatsu) is a fungal plant pathogen specific to *P. lanceolata* L. Like all members of the powdery mildews (Erysiphaceae), it is an obligate pathogen requiring living host tissue throughout its life cycle. Wind-dispersed spores are produced on chains growing vertically on the leaf surface (Braun et al. 2002). During the absence of living host tissue in winter, *P. plantaginis* can survive with the help of specialized resting structures (i.e., chasmothecia). Recent studies have discovered that, unlike in most powdery mildews, resting structures can be produced by selfing (Tollenaere and Laine 2013). Overwintering usually succeeds on only a few plants within the host population in Åland, possibly resulting in strong population bottlenecks between growing seasons (Ovaskainen and Laine 2006).

The host plant *P. lanceolata* (ribwort plantain) is a monoecious, rosette-forming perennial herb (Sagar and Harper 1964). The pollen is wind-dispersed, and *P. lanceolata* is an obligate outcrosser (Ross 1973). Seeds frequently drop close to the mother plant (Bos 1992), and clonal reproduction takes place via the production of side-rosettes from the axillary meristems (Sagar and Harper 1964). Because of clonal reproduction and a seed bank, host populations rarely go extinct, and hence, the spatial configuration of the host populations is relatively constant (Nieminen et al. 2004).

The qualitative interaction (i.e., whether a pathogen strain can infect a host genotype) between *P. plantaginis* and *P. lanceolata* seems characteristic of a gene-for-gene relationship (Thompson and Burdon 1992; Laine 2007). Although the infection intensity (i.e., the degree of infection or damage) is under genetic control (Laine 2007, 2008), the genetic mechanism behind this is yet to be resolved. The powdery mildew lowers plant fitness by extracting resources from the host plant and reducing photosynthesis (Jarvis et al. 2002). Moreover, infection can induce host mortality when infection coincides with other stressful events (Laine 2004).

### PATHOGEN AND HOST MATERIAL

To investigate the impact of spatial scale and habitat configuration on host–parasite coevolution, we collected pathogen strains and plant seeds from nine populations in each of two regions. These regions were chosen to differ strongly in the configuration of the habitat (Fig. 1). In the Åland archipelago in southwestern Finland, the pathogen persists in small host populations ranging from a few square meters to several hectares, with a median size of 300 m^2^. Yearly autumnal surveys conducted in the period 2001–2012 indicate that this highly dynamic pathogen metapopulation persists in the face of high population turnover with approximately half of the pathogen populations going extinct from one year to the next (Laine and Hanski 2006; Tack and Laine, in review). On the island Saaremaa (western Estonia; Fig. 1), the same pathogen occupies large continuous host populations, with the size of the three populations outlined in the course of the current study ranging from 60,200 to 2,560,900 m^2^. The populations in this study were visited in two consecutive years (autumn 2010 and 2011), and no parasite population extinction was observed. This suggests a low frequency of parasite population turnover in Saaremaa.

Given the large number of pathogen strains and plant genotypes, we used a focal/nonfocal design (e.g., Laine 2008), where we inoculated pathogen strains from a subset of populations (i.e., the focal populations) on plant genotypes from both focal and nonfocal populations (Fig 1). This design gives an optimal power to detect local adaptation given a logistically feasible number of replicates. For each island system, we defined three different clusters separated by 6.0–40.0 km. Within each cluster, we selected three populations/locations where the pathogen occurred at a distance of several hundreds of meters up to 1.6 km from each other (Fig. 1). One of these populations was selected as our focal entity, from which we collected four pathogen strains (Fig. 1). We collected seeds from 10 plants from focal populations and five plants from nonfocal populations. Pathogen strains were purified and maintained in the laboratory using methods described in Laine et al. (2006).

### GENETIC PARASITE SAMPLES

From each pathogen population, we sampled a single infected leaf from up to five plants (and in a single population, *n* > 10; Fig. 1). Samples were collected from plants that were located a minimum of one meter apart to better represent genetic diversity within the population. DNA was extracted from the infected leaf samples, and samples were subsequently genotyped using 19 single nucleotide polymorphic (SNP) markers following Tollenaere et al. (2012). The markers allowed for the identification of multilocus genotypes, as characterized by unique combinations of SNP alleles (note that combinations of SNP alleles are henceforth referred to as SNP profile). As the pathogen is haploid, we identified coinfection by the presence of two alleles in at least one SNP locus (Tollenaere et al. 2012).

### INOCULATIONS

To investigate the impact of spatial scale and habitat configuration on the evolutionary outcome of host–parasite interactions, we used a cross-inoculation experiment. Based on our focal/nonfocal design, we crossed each of four pathogen strains from the focal patch with 10 plants from its local population, five plants from each of two populations within the same cluster, five plants from the focal population in each of the two different clusters within the same region, and five plants from each focal population in the other region. The total number of inoculations was then 1076 (see Table S1 for a more detailed overview of the inoculation matrix). At the same time, this design resulted in a set of 24 pathogens (*n* > 4 for each focal population) being inoculated on the same set of 28 host plants. Using this subset of inoculations, we could establish a multihost pathotype for each of these pathogens (i.e., a series of 0/1 values indicating its infectivity on each of the 28 plant genotypes, henceforth referred to as its infectivity profile).

For inoculations, leaves were placed on wet filter paper in Petri dishes and placed in a growth chamber (20 ± 2°C with a 16L/8D photoperiod). Leaves were checked for sporulation on days 7, 8, 9, and 12 (where days 7–9 have been observed as the most common days for the initiation of sporulation; Laine 2007). When infections were first detected on day 12, we conservatively noted the day of first sporulation as day 10. At day 12, when new infections are exceedingly rare, we further assessed the width and length of the largest pathogen colony (which was subsequently converted to colony size presuming an oval shape). Infection intensity (henceforth referred to as aggressiveness) was scored simultaneously on the Bevan scale, where 1 corresponds to sparse mycelium but no conidia; 1.5 to mycelium producing very few conidia and colonies visible under a dissecting microscope; 2.5 to colonies visible with the naked eye but exhibiting sparse sporulation; 3 to profuse sporulation on colonies of moderate size (<5 mm Ø); and 4 to profuse sporulation on large colonies (>5 mm Ø; key adapted from Bevan et al. 1993; Laine 2007). Roughly half of infected leaves (*n* > 342) were checked for the presence/absence of sexual resting structures (chasmothecia or cleistothecia) at day 20, whereas other leaves (*n* > 325) were be discarded for several reasons (e.g., contamination by other microbes).

### FITNESS MEASURES

The interpretation of single pathogen traits as indicators of pathogen fitness may be complicated by genetic or phenotypic correlations (e.g., trade-offs) between pathogen life-history traits. A fitness estimate that is based on multiple life-history stages may account for possible trade-offs in the pathogen life cycle. *Podosphaera plantaginis* genotypes should infect as many host plants as possible within the limited season of spread to enhance their probability of overwintering survival, a critical stage in the life cycle of the pathogen. However, although the pathogen may aim to infect and exploit hosts as fast as possible to increase in numbers, rapid growth may be penalized by the pathogen exhausting its host too rapidly (May and Anderson 1983; Bull 1994; Dybdahl and Storfer 2003). To account for such patterns, we here follow Laine (2008) in calculating two fitness measures, either with or without a penalty for rapidly exhausting the local host. In summary, the fitness measures are derived from sporulation times (*l*) and rates of spore production (*m*) combined to calculate the basic reproductive capacity of powdery mildew strains throughout the growing season. As such, the fitness measure incorporates both the maximum spore production level of the infection, but also the time at which sporulation was initiated. For estimation, it was assumed that a single plant was infected in the beginning of the growing season, under which scenario our fitness measure will directly reflect the cumulative number of plants infected propagating from this source over the entire growing season. This assumes that the density of infected plants remained low enough for the effect of saturation to be neglected, and hence each infectious plant was always assumed to cause *m* new infections each day. This assumption is supported by the results of a study modeling the dynamics of local epidemics in this system which demonstrated the importance of seasonality in this system—conditions become unsuitable for infection development before all available hosts are infected (Ovaskainen and Laine 2006). We then considered two alternative fitness measures. The first measure (“fitness 1” or *f*_1_) assumed that mildew infection does not exhaust the nutrients of the plant, and is able to maintain the maximum spore production level throughout the growing season. The second measure (“fitness 2” or *f*_2_) assumes that nutrient availability in the infected plant is limited, and that spore production will dampen off as the host resources are depleted, resulting in a penalty for exploiting the host too efficiently.

The fitness calculation was implemented through a simple algorithm that kept track of the amount and age of infections throughout the growing season, which was estimated to be 60 days. Denoting by *s_i_*(*t*) the number of plants that were infected *i* days before the current day *t* (in the sense of a spore landing on the plant *i* days ago), the initial condition is given by *s*_0_(1) > 1 and *s*_1_(1) > 0 for *i* > 0, and the aging of the existing infection is described by *s_i_*(*t* + 1) > *s_i_*_−1_(*t*). New infections are initiated due to existing infections that are currently producing spores, so that 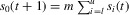 where the upper limit of the summation is *u* > 60 for *f*_1_ and *u* > min(*l* + *d –* 1, 60) for *f*_2_. In both cases, the fitness estimate was calculated as 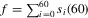. For more details, and for a discussion of the importance of comparing alternative fitness measures, we refer to Laine (2008).

### ANALYSES

We used the framework of generalized linear mixed-effects models (GLMMs; Littell et al. 2006) to analyze the data from the inoculation experiment. Models were fitted with procedure GLIMMIX in SAS 9.3. The framework of generalized linear mixed-effects models is a flexible approach for analyzing univariate data, and has several advantages in the current setting. First, we can specify the distribution of the response variable and link function, which allows us to use the same framework to analyze binomial and normally distributed data (see Table S3 for an overview of response variables, transformations, and link functions). Second, GLMMs allow for the explicit specification of the hierarchical (i.e., nested) design (Fig. 1) to take into account the correlation structure (Littell et al. 2006; Bolker et al. 2009). Third, GLMMs allow making a distinction between fixed and random effects. We model variables as fixed effects when we are interested in specific mean levels, whereas the random effect variables (i) allow for the estimation of variability and (ii) account for the correlation structure within the nested design. Below we provide a brief description of the models used. For a summary of the generalized linear mixed models and more detailed comments on the values reported in the tables, we refer to Appendix A and Tables S2 and S3.

To first assess the relative amount of variation in parasite life-history traits at each spatial scale, we modeled (‘model 1’) each pathogen life-history trait as a function of the random variables “*Pathogen region*”, “*Pathogen population*” (as nested within “*Pathogen region*”), and “*Pathogen genotype*” (as nested within “*Pathogen population*”). As the mean trait level may also depend on variation in the host plant, we further added the random variables “*Host region*,” “*Host population*” (as nested within “*Host region*”), and “*Host genotype*” (as nested within “*Host population*”). To obtain a reasonably balanced and reciprocal data set, we focused on the inoculation data obtained from the inoculations conducted on plant genotypes originating from focal populations. To further investigate differentiation in mean trait levels within the fragmented and continuous regions, we also constructed separate models for Åland and Saaremaa (“models 2 and 3”).

To investigate the spatial scale of local adaptation, we modeled (“model 4”) the fitness traits of the pathogen as a function of the fixed variables “*Pathogen region*” and “*Pathogen population*” (as nested within “*Pathogen region*”). To identify whether there was a consistent impact of distance on the inoculation outcome, we included the fixed categorical variable “*Inoculation type*,” which was coded as: 1 > inoculations among host and pathogen genotypes collected from the same population; 2 > inoculations among host and pathogen genotypes collected from populations within the same cluster; 3 > inoculations among host and pathogen genotypes collected from different clusters but within the same region; and 4 > inoculations among host and pathogen genotypes collected from different regions. Finally, we added the random factors “*Host region*,” “*Host cluster*” (nested within “*Host region*”), “*Host population*” (nested within “*Host cluster*”), and “*Host genotype*” (nested within “*Host population*”) to account for spatial variation in plant resistance. We included the random factor “*Pathogen genotype*” (nested within “*Pathogen population*”) to account for variation among pathogen genotypes. Contrasts based on the factor “*Inoculation type*” were derived to test-specific hypotheses regarding the occurrence and scale of local adaptation (Fig. S1): (i) Are pathogens adapted to local plants (i.e., within-population inoculations) as compared with plants in nearby locations? (ii) Are pathogens adapted to local plants as compared with plants from different clusters in the same region? (iii) Are pathogens adapted to local plants as compared with plants from the other region? iv) Are pathogens adapted to plants in their local cluster as compared to plants in a different cluster within the same region? And (v) are pathogens adapted to plants from their local region as compared to plants from a different region? To further investigate local adaptation within the two regions, we also constructed separate models for Åland and Saaremaa (models 5 and 6).

Finally, we used a multivariate model to investigate pathogen genetic and phenotypic differentiation across multiple spatial scales. The multivariate model was implemented using the function *adonis* in package *vegan* (Oksanen et al. 2013) in *R* 2.15.1 (R Core Team 2012). We note that *adonis*, by partitioning sums of squares of a multivariate data set, is directly analogous to MANOVA (multivariate analysis of variance) and provides an alternative for AMOVA (the nested analysis of molecular variance; Excoffier et al. 1992; Oksanen et al. 2013). Data on SNP profiles and on pathogen infectivity profiles (see sections *Genetic parasite samples* and *Inoculations*; both data sets contain 0/1 data) from focal populations were modeled as a function of “*Pathogen region*” and “*Pathogen population*” (nested within “*Pathogen region*”), where the model residual would represent variation among pathogen strains within populations. As samples with multiple alleles at individual SNP loci are indicative of coinfection by multiple strains (as the pathogen is haploid), these samples were excluded from the multivariate analysis (*n* > 10). We further constructed separate models for each region to test whether the two regions vary in the genetic and phenotypic differentiation among populations.

## Results

### AVERAGE TRAIT VALUES AND TRAIT DIVERSITY

The genetic and phenotypic parasite diversity was remarkably similar among the two regions (Table 1). Based on our 19 SNPs, we detected a total of 33 multilocus genotypes (out of 61 samples), of which three were shared among the two regions. The phenotypic variation among pathogen strains was high, with no strains showing similar responses to all host genotypes. Overall, coinfection was relatively high, with about 29% of the leaf samples containing multiple strains. This fraction was also similar across the two study regions (27.9% and 30.2% in Åland and Saaremaa, respectively).

**Table 1:** Genetic and phenotypic diversity in Åland and Saaremaa

	Overall	Åland	Saaremaa
Coinfection (%)^1^	29.1 (*n* > 86)	27.9 (*n* > 43)	30.2 (*n* > 43)
No. of multilocus genotypes	33 (*n* > 61)	15 (*n* > 31)	21 (*n* > 30)
No. of multihost pathotypes	24 (*n* > 24)	12 (*n* > 12)	12 (*n* > 12)
Genetic diversity^2^	0.72	0.70	0.73
Phenotypic diversity^3^	0.78	0.74	0.81

1 Coinfection is defined as heterogeneity for at least one out of 19 SNPs in a leaf sample.

2 Genetic diversity was calculated as 1 minus the average pair-wise correlation among pathogen strains using SNP profiles.

3 Phenotypic diversity was calculated as 1 minus the average pair-wise correlation among pathogen strains using infectivity profiles.

Strikingly, mean values for parasite life-history traits did not differ among regions (Tables 2 and S4). The spatial scale of differentiation in mean trait values was comparable among Åland and Saaremaa, as most variation was detected among pathogen and host genotypes within populations (Table S5). The results suggest that a larger number of parasite traits were affected by host genotype in Saaremaa, as evidenced by a significant impact of plant genotype on mean trait value for one and six traits in Åland and Saaremaa, respectively (Table S5).

**Table 2:** The spatial scale of variation in mean values of pathogen life-history traits. Shown is the fraction of variation in the mean trait levels explained by each spatial scale. Estimates in bold are significant (*P* < 0.05). For further details, see model 1 in Appendix A

	Pathogen			Host		
Measure (*n*)	Among regions	Among populations	Within populations	Among regions	Among populations	Within populations
Infectivity (*n* > 840)	0.000	0.026	**0.450**	0.000	0.032	**0.492**
Time to sporulation (*n* > 532)	0.000	0.000	**0.255**	0.000	0.002	**0.052**
Aggressiveness (*n* > 527)	0.000	0.000	**0.202**	0.000	0.000	**0.065**
Colony size (*n* > 527)	0.000	0.008	**0.137**	0.000	0.000	**0.058**
Fitness 1 (*n* > 527)	0.000	0.000	**0.240**	0.000	0.000	**0.090**
Fitness 2 (*n* > 527)	0.000	0.000	**0.254**	0.000	0.000	**0.092**
Sexual spore production (*n* > 291)	0.000	0.232	**0.663**	0.001	0.000	0.105

### PATTERNS OF LOCAL ADAPTATION

Contrary to our expectation, there was no sign of local adaptation by the parasite; instead, the parasite was less fit on its local hosts, with parasite maladaptation being most apparent at the large spatial scale (Table 3). Among life-history traits a clear-cut difference emerged in the spatial scale of local adaptation. Both of the measures used to describe pathogen fitness showed signs of parasite maladaptation to plants from the same region (Table 3). In contrast, the production of sexual resting structures was highest on plants inoculated with parasites from different clusters within the same region (Table 3), suggesting parasite maladaptation at the scale of clusters within the two regions.

**Table 3:** Patterns of local adaptation of the powdery mildew *Podosphaera plantaginis* across three spatial scales. Given are least squares means of parasite life-history traits for inoculations among pathogens and plants from the same population and those separated by small, intermediate, and large distances. Of the estimates, “Local scale (1)” refers to inoculations of parasites on plants from the same population, “Small scale (2)” refers to inoculations of parasites on plants from nearby host populations (separated by 0.16–1.6 km), “Intermediate scale (3)” refers to inoculations of parasites on plants from host populations in a different part of the same region (separated by 6–40 km), and “Large-scale (4)” refers to inoculations among the two regions Åland and Saaremaa (set about 200 km apart). Further are reported results from contrasts used to test for patterns of local adaptation across multiple spatial scales (*P* ≤ 0.05 in bold; trend > 0.05 < *P* ≤ 0.10; NS > nonsignificant). For further details on the model and contrasts, see model 4 in Appendix A

	Estimates				Local adaptation contrasts					
Measure (n)	Local scale (1)	Small scale (2)	Intermediate scale (3)	Large-scale (4)	1 vs. 2	1 vs. 3	1 vs. 4	1 + 2 vs. 3	1 + 2 vs. 4	1 + 2 + 3 vs. 4
Infectivity (*n* > 1076)	0.662	0.586	0.711	0.663	NS	NS	NS	NS	NS	NS
Time to sporulation (*n* > 667)	8.275	8.437	8.261	8.174	NS	NS	NS	NS	0.08	0.10
Aggressiveness (*n* > 660)	3.156	3.092	3.196	3.238	NS	NS	NS	NS	**0.04**	0.07
Colony size (*n* > 660)	2.201	2.012	2.356	2.294	NS	NS	NS	**0.03**	0.08	NS
Fitness 1 (*n* > 660)	2.274	2.213	2.327	2.385	NS	NS	0.07	NS	**0.01**	**0.02**
Fitness 2 (*n* > 660)	1.485	1.456	1.506	1.534	NS	NS	0.08	NS	**0.01**	**0.02**
Sexual spore production (*n* > 342)	0.158	0.148	0.285	0.183	NS	0.08	NS	**0.04**	NS	NS

Among the two regions, we detected variation in the spatial scale of local adaptation (Table 4). Although parasite maladaptation to plants from the same cluster was indicated in Åland for three out of seven traits, no such pattern was present in Saaremaa. In Saaremaa, only a single trend (*P* < 0.1) was detected (colony size; as one out of 21 tests), which would be representative of pathogen adaptation at a small spatial scale. However, such a low fraction of significant tests for the continuous region must clearly be interpreted as an absence of any true effect.

**Table 4:** Patterns of local adaptation of the powdery mildew *Podosphaera plantaginis* in Åland and Saaremaa. Given are least squares means of parasite life-history traits for inoculations among pathogens and plants from the same population and those separated by small and intermediate distances. Of the estimates, “Local scale (1)” refers to inoculations of parasites on plants from the same population, “Small scale (2)” refers to inoculations of parasites on plants from nearby host populations (separated by 0.16–1.6 km), and “Intermediate scale (3)” refers to inoculations of parasites on plants from host populations in a different part of the same region (separated by 6–40 km). Further are reported results from contrasts used to test for patterns of local adaptation across multiple spatial scales (*P* ≤ 0.05 in bold; trend > 0.05 < *P* ≤ 0.10; NS > nonsignificant). For further details on the model and contrasts, see models 5 and 6 in Appendix A

		Estimates			Local adaptation contrasts		
Landscape	Measure	Local scale (1)	Small scale (2)	Intermediate scale (3)	1 vs. 2	1 vs. 3	1 + 2 vs. 3
Åland	Infectivity (*n* > 356)	0.650	0.596	0.750	NS	NS	0.09
	Time to sporulation (*n* > 231)	8.209	8.452	8.186	NS	NS	NS
	Aggressiveness (*n* > 226)	3.147	3.170	3.273	NS	NS	**NS**
	Colony size (*n* > 226)	2.146	2.093	2.468	NS	0.10	**0.05**
	Fitness 1 (*n* > 226)	2.251	2.266	2.372	NS	NS	NS
	Fitness 2 (*n* > 226)	1.479	1.478	1.528	NS	NS	NS
	Sexual spore production (*n* > 163)	0.201	0.162	0.350	NS	NS	0.06
Saaremaa	Infectivity (*n* > 360)	0.662	0.556	0.662	NS	NS	NS
	Time to sporulation (*n* > 213)	8.315	8.460	8.353	NS	NS	NS
	Aggressiveness (n > 212)	3.169	3.016	3.100	NS	NS	NS
	Colony size (*n* > 212)	2.228	1.921	2.139	0.09	NS	NS
	Fitness 1 (*n* > 212)	2.318	2.165	2.279	NS	NS	NS
	Fitness 2 (*n* > 212)	1.505	1.437	1.485	NS	NS	NS
	Sexual spore production (*n* > 51)	0.065	0.483	0.183	NS	NS	NS

### COMPARING GENETIC AND PHENOTYPIC DIFFERENTIATION AMONG POPULATIONS

**Figure 2:**
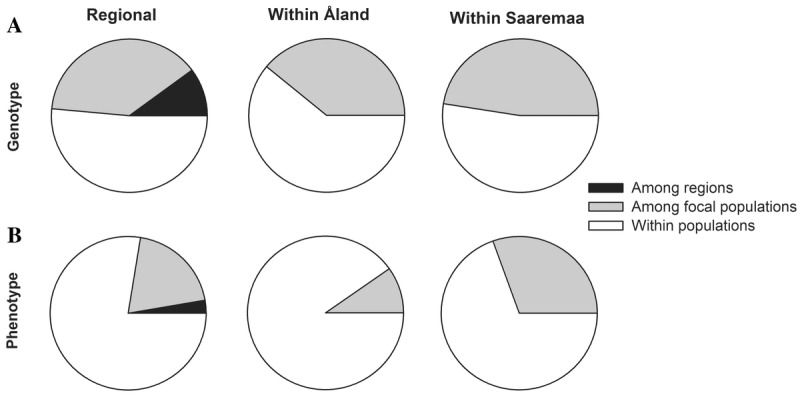
Spatial partitioning of the (A) neutral genetic and (B) phenotypic variation. Genetic variation is based on 19 presumptively neutral SNPs and phenotypic variation is based on the pathogen infection profile on a set of 28 host plants. Left-hand panels refer to results for the full data set, whereas the panels in the middle and on the right refer to region-specific analyses of Åland and Saaremaa, respectively.

Genetic and phenotypic differentiation among populations showed a similar mismatch in both regions (Fig. 2; Table S6): genetic differentiation among populations far exceeds that of phenotypic differentiation among populations on both Åland and Saaremaa. Overall, although there was some genetic differentiation among regions and a considerable amount of genetic differentiation among focal populations in both regions, phenotypic differentiation among populations was much smaller, and nearly absent among regions. The relatively large fraction of phenotypic variation at small spatial scales in both regions supports the prediction that balancing selection maintains phenotypic variation within populations.

## Discussion

Few previous studies have investigated the impact of spatial scale and habitat configuration on coevolutionary dynamics in wild host–parasite systems. The most serious lack relates to studies transferring clear-cut predictions derived in silico to real systems in nature. In this study, we specifically tested a series of explicit hypotheses derived from theory in a single, well-described host–parasite interaction as occurring across variable landscapes. In this context, we made several essential findings.

First, we detected few differences among the two regions in terms of mean parasite trait levels or parasite trait diversity; instead, both regions proved remarkably similar, with most variation in mean trait levels occurring among individual pathogen and plant genotypes within populations. Second, we detected parasite maladaptation among regions, and among populations separated by intermediate distances (6.0–40.0 km) within the fragmented region. Third, in both regions we detected strong genetic differentiation among populations, whereas the majority of phenotypic variation was found within populations. We discuss these findings in further detail in the sections later.

### THE IMPACT OF HABITAT CONFIGURATION ON TRAIT EVOLUTION

Evolutionary epidemiology predicts that host ecology, like spatial structure, may strongly impact on the evolution of parasite traits. Indeed, both theory (Rand et al. 1995; Boots and Sasaki 1999, 2000; Haraguchi and Sasaki 2000; Keeling 2000; van Baalen 2002; Kamo et al. 2007; Lion and van Baalen 2008; Lion and Boots 2010; Best et al. 2011) and microevolutionary selection experiments (Kerr et al. 2006; Boots and Mealor 2007) indicate that virulence, transmission, and trait diversity may evolve in response to habitat configuration. From an applied perspective, such predictions are crucial to understand the long-term consequences of decision-making in natural, agricultural, and human systems (Galvani 2003). For instance, rapid changes in habitat configuration of wild-life habitat (in many cases decreasing connectivity among populations) may select for decreased disease virulence in natural systems (Galvani 2003). The construction of corridors among isolated habitat fragments with the aim to increase population viability of endangered species may have the negative side-effect of increasing selection for virulence in associated diseases. Similarly, the increasing mobility of the human population may increase disease virulence, with major implications for human health (Boots and Sasaki 1999; van Baalen 2002; Galvani 2003). In contrast with these in silico and in vitro predictions, our finding of no or few differences in parasite traits among the two regions suggests that habitat plays a minor role in driving trait diversity in naturam. Alternatively, there may be other factors that counteract or dilute the impact of habitat configuration on trait evolution. If parasite mean traits were largely driven by multiple infections we would not expect any variation in virulence among the two regions, as in our case coinfections were equally common in both regions (Alizon et al. 2013). A major challenge for future investigations may lie in identifying the relative importance of multiple factors in determining parasite trait evolution (e.g., Table 1 in Galvani 2003).

Another notable difference between our findings and those of previous studies may lie in the fact that the *P. lanceolata–P. plantaginis* system is characterized by reciprocal evolutionary dynamics (Laine 2005, 2006; Ovaskainen and Laine 2006; Laine 2008). In contrast, the majority of theoretical explorations and micro- and mesocosm experiments have involved systems where the host did not evolve (Kerr et al. 2006; Lion and Boots 2010). As such, the outcome of coevolutionary interactions may strongly deviate from that expected when only one of the parties is evolving (but see Best et al. 2011).

In summary, we do not find the expected variation in parasite life-history traits among the continuous and fragmented region. Instead, pathogen strains with highly variable infectivity, phenology, and aggressiveness coexist within populations in both regions.

### THE IMPACT OF SPATIAL SCALE AND HABITAT CONFIGURATION ON LOCAL ADAPTATION

The spatial scale of the study has a strong impact on the patterns of local adaptation detected. In the global data set, we mainly detected parasite maladaptation at the scale of the region, indicating a coevolutionary disadvantage of the parasite at this large spatial scale. In the fragmented region, we also detected a weak but consistent sign of parasite maladaptation to plants from the local cluster, as compared to plants from more distant populations within the same region. In contrast, parasite performance did not vary significantly according to host origin in the continuous landscape. As previous studies in this system have shown a mosaic pattern of local adaptation with a tendency for the parasite population to gain the upper hand (Laine 2005, 2008), the current observation of pathogen maladaptation at both an intermediate (in Åland) and large (among the two regions) spatial scale seems surprising. In hindsight, one may argue that it is hard to predict who adapts to whom given the myriad numbers of factors affecting host–parasite coevolutionary dynamics (Greischar and Koskella 2007; Hoeksema and Forde 2008). Such prediction is further complicated by the fact that several of these factors are notoriously difficult to measure empirically (e.g., relative dispersal ability), and there is no straightforward manner to weigh different factors against each other. Importantly, the difference in the perception of who adapts to whom between this and previous studies suggests that the sign of local adaptation may vary in time. Such rapid temporal changes may not be surprising: a recent time-shift experiment by Thrall et al. (2012) demonstrates rapid evolution of flax resistance in response to the local flax rust population. Similarly, previous studies in our pathosystem have indicated that parasite selection pressures can induce rapid and localized increases in plant resistance (Laine 2006; Ovaskainen and Laine 2006). Crucially, such parasite selection pressure on the host plant may show strong temporal fluctuations due to yearly variation in drought stress, which strongly exacerbates parasite selection pressure in this system (Laine 2004). This is in line with a recent study, which shows that environmental conditions may mediate host–parasite coevolution and patterns of local adaptation (Laine 2008). Overall, both spatial and temporal variability in patterns of local adaptation may (partly) explain why two recent reviews have failed to confirm general patterns in terms of the existence or strength of parasite or host local adaptation, or to identify any consistent driving factors determining who adapts to whom in host–parasite interactions (Greischar and Koskella 2007; Hoeksema and Forde 2008).

Given the a posteriori knowledge that the plant here seems ahead in the coevolutionary race, we can reason why there is parasite maladaptation in the fragmented region, and not in the continuous region. Although the plant habitat is, like the pathogen habitat, highly fragmented, there is only minor turnover of plant populations (Nieminen et al. 2004). Hence, the genetic variation and evolutionary potential of plant populations may be high. In contrast, the pathogen faces rapid turnover due to high population extinction rates (Laine and Hanski 2006), which may reduce the evolutionary potential of the pathogen.

In summary, our study establishes the important notion that the existence, sign, and strength of local adaptation may vary with spatial scale, across regions that differ in habitat configuration, and through time. Although complex, such patterns may be essential in explaining the maintenance of phenotypic variation, and fit well with the predictions of the geographic mosaic of coevolution (Thompson 2005; Gandon and Nuismer 2009).

### GENETIC AND PHENOTYPIC DIFFERENTIATION

Although our analysis revealed little genetic differentiation among the two regions, a large fraction of the variation (roughly half) occurred among focal populations within the region. In striking contrast, the majority of the phenotypic variation was found within populations. Such divergence between genetic and phenotypic variation may be explained by balancing selection maintaining phenotypic variation within local populations, whereas limited dispersal and genetic drift result in population differentiation in terms of neutral markers. Our data then support the long-standing theoretical prediction that negative frequency-dependent selection is a major evolutionary force maintaining phenotypic variation within populations (Haldane 1949; Brown and Tellier 2011).

## Conclusion

Micro- and mesocosm experiments have a great tradition in revealing the ecology and evolution of species interactions (Gause 1934; Huffaker 1958; Bohannan and Lenski 2000; Jessup et al. 2004). Such approaches have thus far revealed many fascinating links between spatial structure and parasite evolution (e.g., Kerr et al. 2006; Boots and Mealor 2007). Nonetheless, although such experiments can test theory, reveal biological mechanisms and direct future research, the linkage between micro- and mesocosms and natural communities remains problematic, and this split has recently been reemphasized as one of the major challenges in ecology (Sutherland et al. 2013). Here we took the opposite approach of addressing big questions in the full complexity of a natural system. Naturally, such an approach comes with another set of limitations, the most severe of which relates to the number of replicates achievable. Indeed, although micro- and mesocosms can readily be replicated at the scale of an imaginary metapopulation, such replication is logistically more challenging (or even unfeasible) in natural host–parasite systems. In this study, although we used an optimized design to limit the number of inoculations necessary in the laboratory, we were still limited to comparing a single continuous region with a single fragmented region (cf. Burdon et al. 1999; Carlsson-Granér and Thrall 2002). Nevertheless, we argue that the present type of bold ventures into the natural complexity of real systems may offer the sole solution to ultimately linking theory, small-scale experiments and natural coevolutionary dynamics as playing out in the wild.

In summary, our study highlights the importance of spatial scale and habitat configuration in understanding host–parasite coevolution. Contrary to expectation, we detected a remarkable lack of trait differentiation and diversity among the two regions differing in host configuration, suggesting that factors other than habitat configuration may drive these patterns. Between the two regions we detected local adaptation, and we observed differentiation among the two regions in the strength of local adaptation. Together, these patterns suggest that both spatial scale and habitat configuration may play a key role in understanding coevolutionary outcomes, thereby giving rise to a geographic mosaic of coevolution (Thompson 2005).

## Supporting Information

**Figure S1** Visual depiction of the contrasts specified in models 4–6. **Table S1.** Inoculation matrix using a focal/nonfocal design. **Table S2.** A summary of the generalized linear mixed models fitted for analyses. **Table S3.** The response variables examined in the models described in Table S2. **Table S4.** Mean values and standard deviation of parasite life-history traits for Å land and Saaremaa. **Table S5.** The spatial scale of variation in mean values of pathogen life-history traits for each of two regions. **Table S6.** Spatial partitioning of the neutral genetic and phenotypic variation.
