# Supplementary material for: THE IMPACT OF SPATIAL SCALE AND HABITAT CONFIGURATION ON PATTERNS OF TRAIT VARIATION AND LOCAL ADAPTATION IN A WILD PLANT PARASITE
Source: Evolution. 2013 Sep 11;68(1):176–89. doi: 10.1111/evo.12239 (PMC3916884; doi:10.1111/evo.12239)
Supplement: Supplementary file 1 — Figure S1 Visual depiction of the contrasts specified in models 4–6. Table S1. Inoculation matrix using a focal/nonfocal design. Table S2. A summary of the generalized linear mixed models fitted for analyses. Table S3. The response variables examined in the models described in Table S2. Table S4. Mean values and standard deviation of parasite life-history traits for Å land and Saaremaa. Table S5. The spatial scale of variation in mean values of pathogen life-history traits for each of two regions. Table S6. Spatial partitioning of the neutral genetic and phenotypic variation. [file ev00068-0176-sd1.pdf]

Appendix A. Detailed descriptions of statistical methods.

Overview

As a supplement to the short description of the statistical models provided in the article, we here offer a more detailed description. Statistical models are described verbally below and tabulated in table S2. The response variables, transformations and link functions are identified in Table S3. The contrasts specified to test for local adaptation across multiple scales are depicted in Figure S1.

To analyse the data, we used the framework of generalized linear mixed-effects models (Littell et al. 2006). All models were fitted with procedure GLIMMIX in SAS 9.3.

Models 1, 2 and 3:

To first assess the relative amount of variation in parasite life-history traits (i.e. the mean trait values) across spatial scales, we modelled ('model 1') each pathogen life-history trait as a function of the random variables '*Pathogen region', 'Pathogen population'* (as nested within '*Pathogen region*') and '*Pathogen genotype'* (as nested within '*Pathogen population*'). As the mean trait level may also depend on variation in the host plant, we further added the random variables '*Host region', 'Host population'* (as nested within '*Host region*') and '*Host genotype*' (as nested within '*Host population*'). To obtain a reasonably balanced and reciprocal dataset, we focused on the inoculation data obtained from the inoculations conducted on plant genotypes originating from focal populations. To further investigate differentiation in mean trait levels within the two regions, we also constructed separate models for Åland and Saaremaa. In these models ('models 2 and 3'), we only included the inoculations among pathogen and plant genotypes from either Åland or Saaremaa, respectively.

From these models, we use the parameter estimates of the random effects to calculate the fraction of the total variation explained by each factor. The total variation was calculated as the sum of parameter estimates from the random factors and, in case of a normally distributed response variable (see Table S3), the residual variation. The inherent binomial error was not included in the total variation. Tests for random factors were based on X^2 values (log-likelihood

ratio test with one degree of freedom). The variation explained by each factor and its significance ($p < 0.05$) is reported for model 1 in Table 2 in the main manuscript, and for models 2 and 3 in Table S5.

Models 4, 5 and 6:

To investigate the spatial scale of local adaptation, we modelled ('model 4') the fitness traits of the pathogen as a function of the fixed variables '*Pathogen region*' and '*Pathogen population*' (as nested within '*Pathogen region*'). To identify whether there was a consistent impact of distance on the inoculation outcome we included the fixed categorical variable '*Inoculation type*', which was coded as: 1 = inoculations among host and pathogen genotypes collected from the same population; 2 = inoculations among host and pathogen genotypes collected from populations within the same cluster; 3 = inoculations among host and pathogen genotypes collected from different clusters but within the same region; 4 = inoculations among host and pathogen genotypes collected from different regions. Finally, we added the random factors '*Host region*', '*Host cluster*' (nested within '*Host region*'), '*Host population*' (nested within '*Host cluster*') and '*Host genotype*' (nested within '*Host population*') to account for spatial variation in plant resistance. Finally, we included the random factor '*Pathogen genotype*' (nested within '*Pathogen population*') to account for variation among pathogen genotypes. Contrasts based on the factor '*Inoculation type*' were derived to test specific hypotheses regarding the occurrence and scale of local adaptation (see Fig. S1 for an illustration): i) Are pathogens adapted to local plants (i.e. within-population inoculations) as compared with plants in nearby locations? ii) Are pathogens adapted to local plants as compared with plants from different clusters in the same region? iii) Are pathogens adapted to local plants as compared with plants from the other region? iv) Are pathogens adapted to plants in their local cluster as compared to plants in a different cluster within the same region? And v) are pathogens adapted to plants from their local region as compared to plants from a different region? To further investigate local adaptation within the two regions, we also constructed separate models for Åland and Saaremaa. In these models ('model 5 & 6'), we only included the inoculations among pathogen and plant strains from either Åland or Saaremaa, respectively.

From each model, we extracted the least-squares means for each level of the fixed categorical variable '*Inoculation type*', which are reported in Tables 3 and 4 in the main manuscript. The contrasts identified above were specified using the function lsmestimate in proc GLIMMIX. Significance of the contrasts was evaluated using t-tests.

References

Littell, R. C., G. A. Milliken, W. W. Stroup, R. D. Wolfinger, and O. Schabenberger. 2006. SAS® for Mixed Models. SAS Institute Inc., Cary, USA.

Figure S1. Visual depiction of the contrasts specified in models 4, 5 and 6. Shown are the sampling locations in Åland and Saaremaa. Both regions are drawn at the indicated scale, but the distance between the regions (c. 200 km) is shortened for visual clarity. Shown are – for the westernmost population in Åland – the multiple scales under investigation: (1) refers to inoculations of parasites on plants from the same population, (2) refers to inoculations of parasites on plants from nearby host populations (separated by 0.16 – 1.6 km), (3) refers to inoculations of parasites on plants from host populations in a different part of the same region (6.0 - 40.0 km), and (4) refers to inoculations among the two regions (Åland and Saaremaa) set apart by c. 200 km. Below the graph are the verbal descriptions of contrasts reported in Table 3 in the main manuscript.

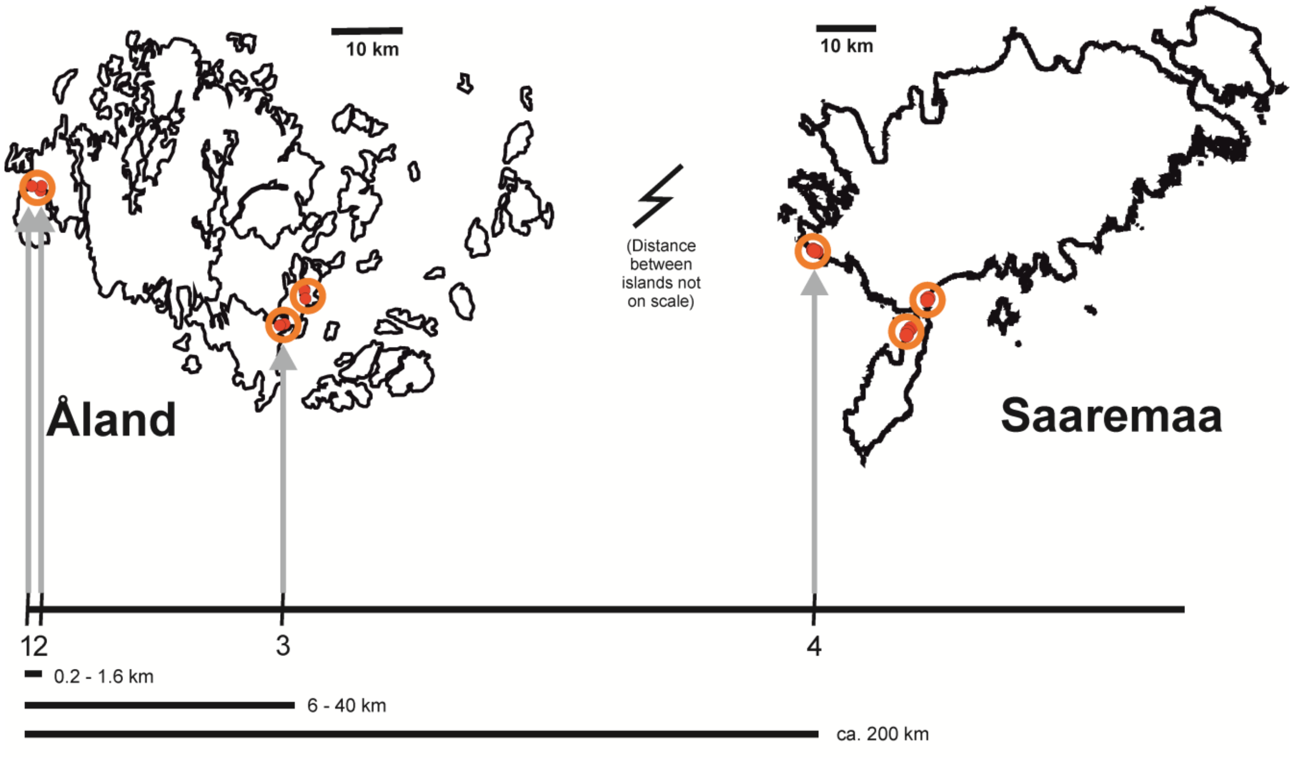

<u>**Contrasts:**</u>
i) 1 *versus* 2: Are pathogens adapted to local plants (i.e. within-population inoculations) as compared with plants in nearby locations?
ii) 1 *versus* 3: Are pathogens adapted to local plants as compared with plants from different clusters on the same island?
iii) 1 *versus* 4: Are pathogens adapted to local plants as compared with plants from the other region?
iv) 1 & 2 *versus* 3: Are pathogens adapted to plants in their local cluster as compared to plants in a different cluster on the same island?
v) 1, 2 & 3 *versus* 4: Are pathogens adapted to plants on their local island as compared to plants on a different island?

Table S1. Inoculation matrix using a focal/non-focal design. For each combination of host and pathogen population are shown the number of inoculations. Each pathogen population was represented by four pathogen strains. The gray scale indicates the distance among host and pathogen populations, ranging from dark grey (inoculations between pathogens and hosts from the same population) to light grey (inoculations among pathogens and hosts from different regions). Focal host populations are in bold.

Host region	Host cluster	Host population	pathogen population 1 (n=4 strains)	pathogen population 4 (n=4 strains)	pathogen population 7 (n=4 strains)	pathogen population 10 (n=4 strains)	pathogen population 13 (n=4 strains)	pathogen population 16 (n=4 strains)
Åland	1	***1***	40	20	20	20	20	20
	1	2	20					
	1	3	16					
	2	***4***	20	40	20	20	20	20
	2	5		20				
	2	6		20				
	3	***7***	20	20	40	20	20	20
	3	8			20			
	3	9			20			
Saaremaa	1	***10***	20	20	20	40	20	20
	1	11				20		
	1	12				20		
	2	***13***	20	20	20	20	40	20
	2	14					20	
	2	15					20	
	3	***16***	20	20	20	20	20	40
	3	17						20
	3	18						20

Table S2. A summary of the generalized linear mixed models fitted for analyses. For each model, we specify the question and spatial scale addressed, the data used, and the fixed and random effects included. For the response variables examined and their link functions, see Table S3.

Model	Question addressed	Largest spatial scale	Inoculation data	Fixed effects	Random effects
**Model 1**	*Do mean parasite traits vary?*	Two regions	Pathogen and host genotypes from focal populations		Pathogen region + Pathogen population (Pathogen region) + Pathogen genotype (Pathogen population) + Host region + Host population (Host region) + Host genotype (Host population)
**Model 2**		Åland	Pathogen and host genotypes from focal populations in Åland		Pathogen population + Pathogen genotype (Pathogen population) + Host population + Host genotype (Host population)
**Model 3**		Saaremaa	Pathogen and host genotypes from focal populations in Saaremaa		Pathogen population + Pathogen genotype (Pathogen population) + Host population + Host genotype (Host population)
**Model 4**	*At which scale do pathogens adapt?*	Two regions	All inoculation data	Pathogen region + Pathogen population (Pathogen region) + Inoculation type	Host region + Host cluster (Host region) + Host population (Host cluster) + Host genotype (Host population) + Pathogen genotype (Pathogen population)
**Model 5**		Åland	Pathogen and host genotypes from populations in Åland	Pathogen population + Inoculation type	Host cluster + Host population (Host cluster) + Host genotype (Host population) + Pathogen genotype (Pathogen population)
**Model 6**		Saaremaa	Pathogen and host genotypes from populations in Saaremaa	Pathogen population + Inoculation type	Host cluster + Host population (Host cluster) + Host genotype (Host population) + Pathogen genotype (Pathogen population)

Table S3. The response variables examined in the models described in Table S2. Given are the response variable, a verbal description, potential transformation and link function used. For identity links, we assumed normally distributed errors and for logit links binomially distributed errors.

Response	Description	Transformation	Link function
Infectivity	Whether or not infection takes place (0/1)		Logit
Time to sporulation	Time to sporulation (days)		Identity
Aggressiveness	Bevan score		Identity
Colony size	Size of the largest colony (mm^2)	Log_{10}	Identity
Fitness 1	Fitness measure (without penalty)		Identity
Fitness 2	Fitness measure (with penalty)		Identity
Sexual spore production	Whether or not sexual spores are present on day 20 (0/1)		Logit

Table S4. Mean values and standard deviation of parasite life-history traits for Åland and Saaremaa. For each region, we used the inoculations between plant and pathogen genotypes collected from the focal populations.

	**Åland**		**Saaremaa**	
	Mean	*Stdev*	*Mean*	*Stdev*
Infectivity	0.675	0.469	0.613	0.488
Time to sporulation	8.043	1.165	8.150	1.213
Aggressiveness	3.255	0.644	3.260	0.649
Colony size	2.415	1.478	2.363	1.046
Fitness 1	2.365	0.673	2.419	0.598
Fitness 2	1.531	0.300	1.549	0.279
Sexual spore production	0.333	0.473	0.162	0.374

Table S5. The spatial scale of variation in mean values of pathogen life-history traits for each of two regions. Shown is the relative amount of variation in the mean trait levels explained by each spatial scale. Estimates in bold are significant ($p<0.05$). For further details, see models 2 and 3 in Appendix A.

	***Measure* (n)**	**Pathogen**		**Host**	
		Among populations	*Within populations*	*Among populations*	*Within populations*
**Åland**	*Infectivity* (n = 240)	0.000	**0.456**	0.179	**0.365**
	Time to sporulation (n = 162)	0.000	**0.274**	0.000	0.017
	Aggressiveness (n = 159)	0.123	**0.166**	0.000	0.062
	Colony size (n = 159)	0.102	**0.199**	0.021	0.055
	Fitness 1 (n = 159)	0.113	**0.185**	0.000	0.061
	Fitness 2 (n = 159)	0.090	**0.202**	0.000	0.068
	Sexual spore production (n = 126)	0.383	**0.591**	0.000	0.026
**Saaremaa**	*Infectivity n =* (240)	0.289	0.161	0.000	**0.549**
	Time to sporulation (n = 147)	0.003	**0.233**	0.020	**0.078**
	Aggressiveness (n = 146)	0.036	**0.258**	0.000	**0.144**
	Colony size (n = 146)	0.071	**0.136**	0.000	**0.150**
	Fitness 1 (n = 146)	0.039	**0.273**	0.000	**0.147**
	Fitness 2 (n = 146)	0.027	**0.264**	0.000	**0.134**
	Sexual spore production (n = 37)	0.000	0.348	0.477	0.174

Table S6. Spatial partitioning of the neutral genetic and phenotypic variation. Genetic variation is based on 19 presumptively neutral SNPs and phenotypic variation is based on the pathogen infection profile on a set of 28 host plants. Shown are R^2-values and p-values.

Data	Region	Among regions	Among populations	Within populations
Genetic	Saaremaa & Åland	0.10 (p = 0.09)	0.39 (p = 0.03)	0.51
	Åland		0.39 (p = 0.09)	0.61
	Saaremaa		0.48 (p = 0.03)	0.52
Phenotypic	Saaremaa & Åland	0.03 (p = 0.65)	0.20 (p = 0.37)	0.78
	Åland		0.10 (p = 0.83)	0.90
	Saaremaa		0.31 (p = 0.05)	0.69
